# Neurological complications of pandemic A(H1N1)2009pdm, postpandemic A(H1N1)v, and seasonal influenza A

**DOI:** 10.1002/brb3.1916

**Published:** 2020-11-05

**Authors:** Daiva Radzišauskienė, Monika Vitkauskaitė, Karolina Žvinytė, Rūta Mameniškienė

**Affiliations:** ^1^ Department of Infectious Diseases and Dermatovenerology Vilnius University Vilnius Lithuania; ^2^ Faculty of Medicine Vilnius University Vilnius Lithuania; ^3^ Department of Neurology and Neurosurgery Vilnius University Vilnius Lithuania

**Keywords:** encephalitis, encephalopathy, influenza, meningitis, polyneuropathy

## Abstract

**Objectives:**

Not much is known about influenza‐associated neurological complications. We aimed to describe the case series of hospitalized patients who were confirmed with influenza A and presented with neurological symptoms in order to capture the broad spectrum of influenza clinical manifestation and suggest including influenza diagnostic in some neurological conditions.

**Materials and methods:**

The inclusion criteria were age ≥ 18 and laboratory‐confirmed influenza presenting with neurological symptoms. Influenza‐associated neurological complication was described as a development of neurological symptom with no other origin. The outcomes were classified into 5 categories: 1. recovery with no significant disability; 2. minor disability (able to manage on their own); 3. moderate disability (requiring some help but able to walk without assistance); 4. severe disability (unable to walk without assistance and perform daily activities); 5. death.

**Results:**

In total, 12 patients (five women and seven men) were enrolled, with age range 18–71 years old. Neurological complications of pandemic A(H_1_N_1_)2009pdm influenza developed in seven out of 69 (10.1%) hospitalized patients. The most common neurological complication was encephalopathy. Neurological complications developed in two out of 24 (8.3%) hospitalized patients during postpandemic (H_1_N_1_)_V_ period. One patient presented with encephalopathy, another with meningoencephalitis. During the 2018 influenza season, there was one patient who has developed influenza A neurological complications. Overall, two out of 104 (1.9%) influenza A patients developed influenza‐associated neurological complications in 2019.

**Conclusions:**

Every patient with unexplained neurological symptoms and signs similar to aseptic and septic meningitis/encephalitis has to be tested for influenza virus during epidemics and pandemics.

## Abstract 2

We present 12 cases of pandemic, post‐pandemic, and seasonal influenza‐associated neurological complications. Our study showed that neurological complications were more common during the pandemic, mostly presenting as encephalopathy and meningoencephalitis within a week of influenza infection. We recommend to test each patient with unexplained neurological symptoms for influenza during the influenza season.

## 1 INTRODUCTION

Influenza is a highly contagious disease caused by RNA viruses of the family *Orthomyxoviridae*. Although influenza affects the respiratory system and most often causes pulmonary complications, it can also cause a variety of neurological complications which are severe enough to require hospitalization. All types of influenza, including seasonal A(H_1_N_1_) and pandemic A(H_1_N_1_)2009pdm influenza, can affect central (CNS) and peripheral (PNS) nervous systems (Blut, 2009; Paksu et al., 2018).

Unlike seasonal influenza, pandemic influenza causes more severe illness, including more frequent neurological complications, in older children and young adults, with only several cases among the elderly. In etiological studies of encephalitis, influenza, both A and B, has been identified in about 10% of cases in children and 8.5% of cases in adults (Studahl, 2003). In comparison, herpes simplex virus (HSV) accounts for approximately 13.8%, Epstein–Barr virus (EBV) for 1.36%, and *Mycobacterium tuberculosis* for 5% of encephalitis cases (Gnann and Whitley, 2017; Granerod et al., 2010; Kumar et al., 2018). Neurological complications of pandemic influenza A (H_1_N_1_)2009pdm have been reported in 6%–10% of pediatric cases (Takkar et al., 2015). Since neurological complications are rare, there is a lack of published studies covering this topic. However, smaller studies and case reports describe such complications as Guillain–Barre syndrome (GBS), transverse myelitis, meningoencephalitis, and, the most troubling complication, encephalopathy or encephalitis, which might rapidly evolve to an acute necrotic encephalitis, causing lesions in thalami and cerebral cortex (Ambrozaitis et al., 2016; Davis, 2010; Rothberg & Haessler, 2010). Severe encephalitis can cause lethargy, coma, or even death of the patient (Ferrari et al., 2009). The pathogenesis of neurological complications of influenza is not fully understood, and diagnostic methods are not standardized.

The aim of this study is to describe the cases of pandemic A(H_1_N_1_)2009pdm, postpandemic (H_1_N_1_)_V,_ and seasonal influenza A presented with neurological manifestation in order to help capture the wide spectrum of influenza clinical presentation and propose to include influenza diagnostic in some neurological conditions.

## 2 METHODS

### 2.1 Study population

The data of 2 studies, carried out during pandemic A(H_1_N_1_)2009pdm, postpandemic (H_1_N_1_)_V_ (2009–2011), and seasonal (2018–2019) influenza periods, were used. These studies were performed in Infectious Diseases Center of Vilnius University Hospital Santaros Klinikos, which is the reference center for adult infectious diseases in Vilnius district. It serves the population of 809,000, which is 27% of the nation's population (Ambrozaitis et al., 2016). In total, 12 patients who presented with neurological influenza complications were included for analysis. The inclusion criteria were age ≥ 18 and laboratory‐confirmed influenza manifesting with neurological complications.

### 2.2 Data collection

The demographic and clinical characteristic of the patients were recorded. The variables for the characterization of the cases were age, sex, traveling history, exposure, underlying conditions, vaccination status, clinical presentations, dates (onset of symptoms, manifestation of first neurological symptom, treatment started), laboratory tests, and outcomes.

### 2.3 Data availability

The data that support the findings of this study are available on request from the corresponding author. The data are not publicly available due to privacy or ethical restrictions.

### 2.4 Diagnosis and definitions

During the pandemic and postpandemic periods in 2009–2011, real‐time reverse transcriptase polymerase chain reaction (rRT‐PCR) was performed using assay kits provided by the World Health Organization (WHO). Testing was performed at the National Public Health Surveillance Laboratory of Lithuania. During seasonal influenza periods in 2018–2019, testing was performed in local laboratory of Vilnius University Hospital Santaros Klinikos. Automated, multiplex, real‐time reverse transcriptase polymerase chain reaction (rRT‐PCR) assay was used for the in vitro qualitative detection and differentiation of influenza A, influenza B, and respiratory syncytial virus (RSV) viral RNA. The influenza A viruses were not sub‐typed. The samples of cerebrospinal fluid (CSF) of all patients were cultured for bacteria and tested for other viral neurological infections. All patients were tested for human immunodeficiency virus (HIV).

An influenza‐related neurological complication was defined as the emergence of neurological symptom with no explanation of other etiology. Encephalitis was defined as the presence of parenchymatous brain involvement signs such as focal neurological signs, seizures, decreased consciousness, and delirium concomitant with CSF leukocytes >5 × 10^6^/L. Encephalopathy was defined as the presence of signs listed above with normal leukocytes count in CSF. Meningitis was defined as CSF leukocytes >5 × 10^6^/L.

### 2.5 Classification of outcomes

The outcomes were classified into 5 categories: 1. recovery with no significant disability; 2. minor disability (able to manage on their own); 3. moderate disability (requiring some help, but able to walk without assistance); 4. severe disability (unable to walk without assistance and perform daily activities); 5. death.

### 2.6 Statistical analysis

Descriptive statistics were used for data analysis. Vilnius Regional Bioethics Committee approved the pandemic A(H_1_N_1_)2009pdm and postpandemic (H_1_N_1_)_V_) study in 2009–2011 protocol; this was deemed as a minimal risk, and a written consent was waived. Since 2018, a study “Etiology and sequelae of infectious encephalitis” has been started in Infectious Diseases Center of Vilnius University Hospital Santaros Klinikos. Vilnius Regional Bioethics Committee approved this study protocol.

## 3 RESULTS

A total number of 12 patients with neurological complications of influenza A virus infection were included in this study. The majority of the study participants were young (median age: 27, range: 18–71 years old). The baseline study characteristics are summarized in Table 1. None of the patients were vaccinated with either seasonal or pandemic influenza vaccine, also none of them were vaccinated with pneumococcal vaccine, and they did not receive anti‐influenza antiviral therapy on an outpatient basis. None of the patients had left Lithuania in the period of 1 month before becoming ill. Everyone was HIV‐negative.

**Table 1:** Main characteristics of patients with pandemic influenza A(H_1_N_1_) 2009pdm and patients with neurological influenza A complications

Study characteristic	Patient group		
Demographic characteristics	All patients with neurological Influenza A complications (*n* = 12)	Pandemic influenza A(H_1_N_1_) 2009pdm patients with neurological complications (*n* = 7)	All pandemic influenza A(H_1_N_1_) 2009pdm patients (*n* = 69)
Sex, male, number (%)	5 (41.7)	3 (42.9)	33 (47.8)
Sex, female, number (%)	7 (58.3)	4 (57.1)	36 (52.2)
Age, min‐max, years	18–71	18–52	18–67
Age, median, years	27	21	25
Epidemiological characteristics			
Reported exposure, number (%):	4 (33.3)	4 (57.1)	44 (63.8)
Infected at work or in an educational institution, number (%)	2 (16.7)	2 (28.6)	24 (34.8)
Infected in their household, number (%)	2 (16.7)	2 (28.6)	20 (29.0)
Unknown, number (%)	8 (66.7)	3 (42.9)	25 (36.2)
Length of hospital stay (days)			
Median (min‐max)	10 (3–37)	6 (3–37)	6 (2–37)
Admitted to intense care unit, number (%)	9 (75.0)	6 (85.7)	9 (13.0)
Length of intense care unit stay (days)			
Median (min‐max)	1 (1–17)	1.5 (1–17)	2 (1–37)
Pre‐existing conditions			
No evidence of pre‐existing conditions, number (%)	6 (50.0)	4 (57.1)	49 (71.0)
Pre‐existing conditions, number (%)	6 (50.0)	3 (42.9)	20 (29.0)
Type 2 diabetes mellitus, number (%)	2 (16.7)	1 (14.3)	3 (4.3)
Chronic CNS diseases, number (%)	2 (16.7)	2 (28.6)	6 (8.7)
Cardiovascular diseases, number (%)	1 (8.3)	0	2 (2.9)
Psoriasis, number (%)	1 (8.3)	0	0
COPD, number (%)	0	0	5 (7.2)

### 3.1 Pandemic influenza

The peak of influenza A(H_1_N_1_)2009pdm pandemic in Lithuania started on 1 November, 2009, and lasted until 31 January, 2010. During this period, 69 patients with influenza A(H_1_N_1_)2009pdm and 21 patient with seasonal influenza A(H_1_N_1_) were treated in Infectious Diseases Center. Neither influenza A(H_2_N_3_), nor influenza B was diagnosed. Neurological complications developed only in hospitalized patients with pandemic A(H_1_N_1_)2009pdm (7/69 (10.1%) of patients). The most common complication was influenza encephalopathy (Cases 1–5, Table 2); other patients were diagnosed with bacterial meningitis (Case 6) and tetraparesis (Case 7). Patients diagnosed with encephalopathy (Cases 1–5, Table 2) were young, from 18 to 27 years old, and most of them had no comorbidities, except one patient with previous head trauma and epilepsy. Four patients out of seven were students. The patients presented with altered consciousness (Glasgow coma scale (GCS) 9–13), which lasted 1–2 days (Cases 1–5, Table 2). One patient developed bacterial meningitis (case 6), the other one delirium and polyneuropathy (case 7).

**Table 2:** Clinical, laboratory, and imaging findings of patients with Influenza A‐related neurological complications

Case	Age, sex	Comorbidity	Influenza subtype/year	Neurological symptoms	Day of illness, when neurological symptoms developed	Diagnosis	CSF	Blood analysis, CRP	Head CT/MRT	Days in ICU/at hospital	Treatment regimen	Outcome/Rankin scale
1	27, F	None	A(H_1_N_1_) 2009pdm	Somnolence for 2 days. GCS 13	3	Pandemic influenza H_1_N_1_ encephalopathy	Not done	WBC 10 × 10^9^, band NEU – 32%, PLT 143 × 10^9^/L, ESR 12 mm/h, CRP 109 mg/L > 192 mg/L at 3rd day of hospitalization	Not done	0/8	75 mg of oseltamivir twice daily for 5 days	Full recovery/1
2	21, F	None	A(H_1_N_1_) 2009pdm	Stupor for 1 day. GCS 9	2	Pandemic influenza H_1_N_1_ encephalopathy	Normal. Culture negative. PCR for HSV, EBV, HHV6,7 negative	WBC 27 × 10^9^/L, band NEU – 39%, RBC 3.61 × 10^12^/L and Hb 86 g/L at 15th day of hospitalization, ESR 55 mm/h, CPR 450 mg/L, Creatinine 402 µmol/L	Not done	1/6	75 mg of oseltamivir twice daily for 5 days	Full recovery/1
3	18, M	None	A(H_1_N_1_) 2009pdm	Somnolence for 2 days. GCS 13	2	Pandemic influenza H_1_N_1_ encephalopathy	Not done	Normal	Not done	2/5	75 mg of oseltamivir twice daily for 5 days	Full recovery/1
4	18, F	Head trauma 5 years ago	A(H_1_N_1_) 2009pdm	Somnolence for 1 day. GCS 13	2	Pandemic influenza H_1_N_1_ encephalopathy	Normal	Normal	Not done	1/3	75 mg of oseltamivir twice daily for 5 days	Full recovery/1
5	20, F	None	A(H_1_N_1_) 2009pdm	Somnolence for 1 day. GCS 13	3	Pandemic influenza H_1_N_1_ encephalopathy	Normal	Normal	Not done	1/3	75 mg of oseltamivir twice daily for 5 days	Full recovery/1
6	24, M	Symptomatic post‐traumatic epilepsy	A(H_1_N_1_) 2009pdm	Seizures. Comma GCS 8	5	Bacterial meningitis	**Day 1** – cytosis 240/mm^3^, NEU 85%, PRO 9.2 g/L, GLUC 0.3 mmol/L **Day 4 ‐** cytosis 907/mm^3^, NEU 95%, PRO 4.5 g/L, GLUC 2.9 mmol/L **Day 19 ‐** cytosis 150/mm^3^, LYM 96%, PRO 0.8 g/L, GLUC 2.1 mmol/L	WBC 3.77 × 10^9^, CRP normal	CT normal	14/22	150 mg of oseltamivir twice daily for 5 days, then 75mg for 5 more days	Minor disability/2
7	52, M	Diabetes mellitus	A(H_1_N_1_) 2009pdm	Flaccid tetraparesis with sensory polyneuropathy	19	Delirium Distal sensory polyneuropathy Tetraparesis	Normal	Hb 104 g/L, CRP normal	Not done	17/37	150 mg of oseltamivir twice daily for 5 days, then 75mg for 5 more days	Severe disability/4
8	41, F	None	Postpandemic (H_1_N_1_)_V_ 2011	Stupor. GCS 12	2	Influenza A(H_1_N_1_) Encephalopathy	Normal	Normal	Not done	0/5	75 mg of oseltamivir twice daily for 5 days	Full recovery/1
9	25, M	None	Postpandemic (H_1_N_1_)_V_ 2011	Headache, vomiting, coordination disorder	5	Influenza A(H_1_N_1_) meningoencephalitis	Cytosis 347/mm^3^, LYM 100%, PRO 1.5 g/L, GLUC 2.6 mmol/L	Normal	CT ‐ normal	0/18	75 mg of oseltamivir twice daily for 5 days	Minor disability/2
10	58, M	Subarachnoid post‐traumatic hemorrhage in 2015, liquorrhea	Seasonal influenza A 2018	Confusion, behavior disorders, disorientation, language disorders, coordination disorders	14	Meningoencephalitis of unknown etiology	**Day 1 ‐** cytosis 320/mm^3^, NEU 76%, LYM 24%, PRO 0.8 g/L, GLUC 2.8 mmol/L **Day 19 –** normal	WBC 18 × 10^9^/L, NEU 13 × 10^9^/L, neutrophils with toxic granulation 1 point, CRP 274 mg/L	**Day 7 –**meninges accumulate contrast, old post‐traumatic lesions in left frontal lobe **Day 19 ‐** contrast accumulation is less pronounce, leak in *lamina cribrosa*	3/26	150 mg of oseltamivir twice daily for 3 days, than 75mg twice daily for more 5 days	Minor disability/2
11	33, M	Psoriasis	Seasonal influenza A 2019	Confusion, behavior disorders, disorientation, language disorders coordination disorders	6	Influenza A(H_1_N_1_) meningoencephalitis	Cytosis 80/mm^3^, LYM 99%, PRO 1.1 g/L, GLUC 2.8 mmol/L	Normal	CT normal	1/12	75 mg of oseltamivir twice daily for 5 days	Moderate disability/3
12	71, M	Diabetes mellitus, arterial hypertension	Seasonal influenza A 2019	Confusion, behavior, language, memory, coordination disorders, disorientation, nystagmus, positive Kernig's sign	7	Influenza A(H_1_N_1_) meningoencephalitis	Cytosis 348/mm^3^, LYM 96%, PRO 1.2 g/L, GLUC 5.2 mmol/L	WBC 3 × 10^9^/L, NEU 2 × 10^9^/L, PLT 133 × 10^9^/L, CRP normal	CT normal	1/15	75 mg of oseltamivir twice daily for 5 days	Moderate disability/3

Note

CRP – C‐reactive protein, CSF – cerebrospinal fluid, CT – computerized tomography, ESR – erythrocyte sedimentation rate, F – female, GCS – Glasgow coma scale, GLUC – glucose, Hb – hemoglobin, ICU – intensive care unit, LYM – lymphocytes, M‐ male, MRI – magnetic resonance imaging, NEU – neutrophils, PCR – polymerase chain reaction, PLT – platelet count, PRO – protein, RBC – red blood cell count, WBC – white blood cell count.

#### 3.1.1 Case 6

A 24‐year‐old patient was hospitalized on day 5 of the disease onset, with suspected bacterial meningitis. The disease started with a fever up to 39°C, headache, and productive cough. On the day of the arrival to the emergency unit, he complained of having a very severe headache. During the examination, he had meningeal symptoms, including neck muscle rigidity, Kernig's and Brudzinski's, hemorrhagic rash on the chest. 50 min later, he had an epileptic seizure, which was interrupted with intravenous injection of 10 mg diazepam. After 25 min, the seizure recurred and the patient developed status epilepticus. The patient was transferred to the intensive care unit. Blood and cerebrospinal fluid analysis showed signs for bacterial meningitis (Table 2). Pandemic A(H_1_N_1_)2009pdm influenza virus has been detected from nose and throat swabs. Patient's treatment included intravenous ceftriaxone and oral oseltamivir 150 mg 2 times per day administration. The next day, patient's condition has worsened, and he developed a coma of GCS 8 points and shortness of breathing. Chest X‐ray showed bilateral pneumonia. Vancomycin was additionally prescribed. On the fourth day of hospitalization, the patient was intubated. Imipenem/cilastatin was prescribed. Repeated CSF analysis showed decrease in protein and normal glucose levels. C‐reactive protein (CRP) decreased to 88 mg/L. On the fifth day of hospitalization, the patient developed pneumothorax and was connected to the Bobrov's machine. The patient was ventilated for 5 days and regained consciousness after 7 days. Bobrov's machine functioned for 10 days period. Blood, CSF, and tracheal aspirate cultures were negative. The patient was discharged from the hospital with minor disabilities, including general weakness and intermittent headache.

#### 3.1.2 Case 7

A 52‐year‐old man with a history of diabetes mellitus was admitted to the intensive care unit on the fifth day from the onset of the disease. Patient was an active worker. He reported having had a contact with influenza virus‐infected people at work. He presented with a fever up to 39°C, productive cough, and shortness of breath. Respiratory failure requiring mechanical ventilation has developed. Blood test revealed: white blood cell count (WBC) 27 × 10^9^/L, band neutrophils 27% of WBC, CRP 450 mg/L, also, chest X‐ray showed bilateral pneumonia. On the sixth day of hospitalization, kidney failure developed (serum creatinine – 402 µmol/L) and anemia occurred (hemoglobin (Hb) 86 g/L). Blood, cerebrospinal fluid, and urine cultures were negative, while *S. aureus* growth was found in endotracheal aspirate sample. Artificial lung ventilation was applied for 13 days. On the fourteenth day after admission to the intensive care unit (ICU), delirium, distal sensomotor polyneuropathy, and tetraparesis developed. Lumbar puncture was performed but the CSF analysis showed no abnormalities. Patient was transferred to the nursing hospital with severe residual phenomena: tetraparesis, cognitive impairment, and a score of 25 on the Barthel index.

### 3.2 Postpandemic (H_1_N_1_)_V_ influenza

Postpandemic (H_1_N_1_)_V_ influenza season in Lithuania started on 1 January, 2011, and lasted until 15 May, 2011. A total of 24 patients with influenza (H_1_N_1_)_V_ and 3 patients with influenza B were treated in Infectious Diseases Center during this period. The mean age of influenza (H_1_N_1_)v patients was 31.4 years (range: 18–70; median age: 28.5); 13 (54.2%) patients were male. Two patients out of 24 (8.3%) were admitted to the intense care unit (ICU), and median length of ICU stay was 2.5 days (range: 1–4). Reported exposure was in 15 (62.5%) of confirmed cases. Neurological complications developed in two out of 24 (8.3%) hospitalized patients with influenza A (H_1_N_1_)v. No neurological complications were observed in influenza B patients. One patient presented with encephalopathy, another with meningoencephalitis (Cases 8–9, Table 2). Both of them had no comorbidities. Neurological symptoms for the patient with encephalopathy manifested on the second day, whereas the patient with meningoencephalitis had them develop on the fifth day of the disease (Table 2). The patient with meningoencephalitis was hospitalized for a longer period of 18 days, compared to the other patient who was hospitalized for 5 days. Patient with meningoencephalitis was discharged with a minor disability: ataxia (Table 2).

### 3.3 Seasonal influenza A

A total of 197 patients with influenza were treated in the Infectious diseases Center during the 2018 influenza season. The mean age was 60.4 years (range: 19–97; median age: 64). A total of 116 (58.9%) patients were women. Influenza B dominated this season and accounted for 176/197 (89.3%) cases of influenza. It is noteworthy to mention that none of the influenza B patients developed neurological complications. One patient with influenza A out of 21 (4.7%) has developed neurological complications.

In total, two out of 104 (1.9%) patients diagnosed with influenza A have developed neurological complications in 2019. During this season, there were no confirmed cases of influenza B. The mean age of patients with influenza A was 60.5 years (range: 19–97; median age: 65). A total of 51 (49.0%) patients were women.

#### 3.3.1 Case 10

A 58‐year‐old patient presented with fever up to 39°C, headache, and dizziness in March 2018. He has had no contact with influenza virus‐infected people. The patient is a construction worker; in 2015, he had had a head trauma at work, and a surgery for subarachnoid hemorrhage was performed with no neurological consequences; however, watery secretion from nose was initiated. The patient was a smoker. Anti‐influenza antiviral medicine and antibiotics were not prescribed in outpatient settings. Two weeks later, the patient became disoriented and had behavioral changes, speech, and coordination impairment. He was admitted to the intensive care unit. CSF analysis showed lymphocytic pleocytosis (320 × 10^6^), moderate elevation of protein, and normal concentration of glucose; also, blood test showed signs of bacterial infection (Table 2). Nose and throat swabs confirmed influenza A. rRT‐PCR assay on CSF for herpes simplex virus (HSV), human herpes virus 6 (HHV‐6), HHV‐7, HHV‐8 DNA was performed. There was no bacterial growth in both the blood and CSF cultures. *N. meningitidis, H. influenzae, S. pneumoniae, L. monocytogenes, S. β haemolyticus* DNA were not detected by rRT‐PCR assay on CSF. After 3 days, both the state of consciousness and language recovered. A head magnetic resonance imaging (MRI) done the following week showed signs of meningitis: accumulation of contrast, increased diffusion restriction in left parietal and bilateral frontal, thickening of the mucosa of the left frontal sinus and ethmoidal cells, post‐traumatic deformation of the left frontal sinus, and post‐traumatic encephalomalacia in the left frontal lobe. 2 weeks later, head MRI was repeated and showed a reduction of both contrast accumulation in the meninges and the diffuse restriction; thickened mucosa of the left frontal sinus accumulated the contrast more intensively, focal point of encephalomalacia showed no dynamics. A defect was found in the dorsal wall of the left frontal bone with a slight intracranial accumulation and a defect in the lamina cribrosa was also observed.

The patient was treated with oseltamivir 150 mg twice a day for 3 days and 75 mg twice a day for 5 days with a short course of intravenous acyclovir, as well as antibiotics such as ceftriaxone and ampicillin. He was transferred to neurosurgery unit for cranioplasty with minor disability.

#### 3.3.2 Cases 11, 12

In 2019, two patients developed neurological influenza complication—meningoencephalitis. One patient (case 11) was 33 years old, and the other one (case 12) was 71. Both of them became ill in January. The older patient had an epidemiological history of a contact with a flu‐sick grandchild. Both patients had similar clinical symptoms, including disorientation, speech, behavior, and coordination disorders. HSV, varicella zoster virus (VZV), HHV‐6, HHV‐7, HHV‐8 DNA, and RNA of enteroviruses were not found in CSF. Tick‐borne encephalitis virus (TBEV) antibody testing for IgM and IgG in serum was negative. Blood and CSF cultures were negative. Patients were treated with oseltamivir 75 mg two times per day orally. Both patients were discharged to a rehabilitation unit with moderate residual events such as headache, dizziness, general weakness, and coordination disorders. For residual events, both patients will be followed up for 5 more years. 3 months after discharge, patient 11 felt fatigue, headache, and general weakness. Electroencephalography (EEG) test results were without pathology 6 months after discharge, Moca cognitive test was 26/30, and there were no coordination disorders. Patient 12 complained having a coordination disorder 6 months after discharge with minor evidence of cerebellar ataxia. This patient's EEG was registered without pathology and Moca test was 27/30.

## 4 DISCUSSION

Neurological manifestations of influenza were first reported in 1918 (Goenka et al., 2014). The incidence of influenza neurological complications has increased after 2009 H_1_N_1_ pandemic (Meijer et al., 2016). It has been found that neurological complications appear to 0.21/1,000,000 symptomatic patients per year (Hjalmarsson et al., 2009). A study of influenza‐related neurological complications in the USA discovered a higher incidence rate in Asian/Pacific Islands population (12.79/1,000,000) compared to non‐Hispanic/white patients (3.09/1,000,000), implying a plausible genetic predisposition (Sellers et al., 2017). Moreover, in Japan, the most commonly identified pathogen causing an acute encephalopathy is influenza virus (Goenka et al., 2014). According to Lee et al., neurological complications appear to be more common in patients with pandemic influenza (6.6%) than those with seasonal influenza (4.5%) (Lee et al., 2011). In total, 59,000 influenza cases were reported during the peak of influenza A(H_1_N_1_)2009pdm pandemic in Lithuania (Center for Communicable Diseases & AIDS). This represents 0.021% (59,000/2.8 million) of the Lithuanian population. The highest incidence of influenza (580 cases per 100,000 people) was reported during week 48 (Center for Communicable Diseases & AIDS). The laboratory‐confirmed pandemic influenza occurred in 810 patients during the peak of pandemic. During 2011 postpandemic influenza season, a total of 41,849 influenza cases were reported in Lithuania. Influenza A(H_1_N_1_)_V_ dominated this season and was detected in 67% of samples (Center for Communicable Diseases & AIDS). In 2018 influenza season, a total of 57,759 influenza cases were reported in Lithuania. The influenza B virus (85.8%), like in majority of the Europe (63%), dominated this season in Lithuania (Center for Communicable Diseases & AIDS). A total of 49,661 cases of influenza were registered in 2019. Virus A dominated this season: Virus A was detected in 1,747 (99.4%) cases, and virus B in 4 (0.2%) cases out of 1,758 samples at the National Public Health Surveillance Laboratory of Lithuania (Center for Communicable Diseases & AIDS). Influenza A(H_1_)v accounted for 43% of all confirmed influenza A type virus, influenza A(H_3_) for 52.5%, and influenza (H_1_) for 3.3% (Center for Communicable Diseases & AIDS). Consistent to this study, we found that two patients out of 104 hospitalized with confirmed seasonal influenza A (1.9%) developed neurological complications compared to seven patients out of 69 (10.1%) with confirmed pandemic A(H_1_N_1_)2009pdm influenza. Pandemic strains can suppress the expression of over 30 genes involved in neuronal gene networks, suggesting a possible predisposition of the host neurologic complications (Bengualid & Berger, 2017). The neurological complications of influenza B are considered to be rather mild than severe and less frequent compared to influenza A; for example, influenza B accounts for approximately 10% of influenza‐associated encephalopathy (IAE) (Popescu et al., 2017; Surtees & DeSousa, 2006). This might be due to a slower mutation rate compared to influenza A. We had no cases of influenza B neurological complications in our study, although in 2018, 176 patients with influenza B were treated. However, there is a lack of extensive studies on the neurological complications of influenza B (Moon et al., 2013).

Neurological complications of influenza can manifest with a variety of syndromes; mostly reported include seizures, encephalopathy, meningoencephalitis, transverse myelitis, GBS, acute disseminated encephalomyelitis, Reye syndrome, speech and motor disorders, and extrapyramidal syndromes, such as dystonia and chorea (Kumar et al., 2018; Meijer et al., 2016; Paksu et al., 2018). In our study, neurological influenza complications developed more often during the pandemic. Young patients (age 18–27, except one 52‐year‐old patient) were affected. The majority of them (five out of seven) had no comorbidities. The two patients with comorbidities (diabetes mellitus and post‐traumatic epilepsy) developed more serious neurological complications. The most common complication was encephalopathy. IAE is initially characterized by altered level of consciousness appearing within a few days of influenza infection (Sellers et al., 2017). A variety of clinical syndromes are associated with IAE, including acute necrotizing encephalopathy, acute encephalopathy with biphasic seizures and late restricted diffusion, and mild encephalopathy with reversible splenial lesion (Ferrari et al., 2009; Hjalmarsson et al., 2009). In our study, patients with encephalopathy presented with short‐lasting stupor or coma. All of them have completely recovered.

There is no single mechanism which would explain the origin of neurological complications of influenza. Theories include immune response susceptibility (cytokine storm theory), influenza virus invasion to CNS, postinfectious immune‐mediated process, genetic factors, *RANBP2* gene mutations (Goenka et al., 2014; Meijer et al., 2016; Sellers et al., 2017; Takkar et al., 2015). According to cytokine storm theory, respiratory viral infection provokes pro‐inflammatory cytokines that travel to the brain through blood (Takkar et al., 2015). This theory is supported by significantly elevated levels of serum cytokines IL‐6, TNF‐alpha, and IL‐10 in patients with IAE compared to those with no neurological disorders or not infected with influenza virus. The correlation between the severity of neurological complications and serum levels of pro‐inflammatory cytokines also supports the hypothesis of a cytokine driven mechanism (Algahtani & Shirah, 2016; Hasegawa et al., 2011; Kawada et al., 2003; Sellers et al., 2017). Pro‐inflammatory cytokines may increase the permeability of hematoencephalic barrier, trigger an injury of vascular endothelium as well as apoptosis of neurons and glia cells, and provoke acute brain edema and necrosis. Prolonged activation of microglia may damage neurons and impair synaptic transmission and structure (Hosseini et al., 2018; Ito et al., 1999).

Initially, influenza viruses do not show a direct tropism to the nervous system. However, virus detection in retina, the olfactory bulb, and trigeminal nerve has been described in animal models and may be favored by the free nerve endings near the influenza‐infected epithelial cells in the upper respiratory tract (Cárdenas et al., 2014; Studahl, 2003). Additionally, there are data on influenza virus being found on brain tissue in neuropil, ependymal, Purkinje cells, other neurons, and cerebrospinal fluid (Cárdenas et al., 2014). *Tomonaga et al*. described that neurotropic strains of influenza A virus may enter the CNS through the hematoencephalic barrier and vascular endothelial cells (Tomonaga, 2004). Viral RNA in brain tissue and CSF implies a direct viral invasion of the CNS (Sellers et al., 2017; Steininger et al., 2003). In our study, the influenza encephalopathy was most likely associated with an increase of pro‐inflammatory cytokines in blood, as direct influenza virus‐induced neuronal damage would lead to a more severe presentation. The patients presented as early as 2–3 days since the onset of illness and all of them were treated with oseltamivir immediately after hospitalization.

Another theory suggesting that neurological manifestations are secondary to a postinfectious immune‐mediated process in the CNS is based on the absence of the virus RNA in the CSF according to studies (Meijer et al., 2016; Sellers et al., 2017). GBS, acute disseminated encephalomyelitis, and transverse myelitis are immune‐mediated complications of influenza. Molecular mimicry of influenza virus epitopes and normal human brain antigens is yet to be discovered (Lei et al., 2012; Sellers et al., 2017). A Canadian study demonstrated a lower risk of GBS in individuals vaccinated with trivalent inactivated influenza vaccine compared to unvaccinated individuals (Kwong et al., 2013). We hypothesize that in our study, polyneuropathy was associated with postinfectious immune mechanisms. The neurological complications developed late on the 19th day of illness.

Bacterial co‐infection is observed in up to 65% of adult influenza cases (Goenka et al., 2014; Klein et al., 2016). It is proposed that influenza virus could damage the epithelial lining of the respiratory tract by causing epithelial cell death and degrading mucins in addition to virus‐mediated dysfunction of neutrophils and macrophages, allowing bacterial agents to foothold (McCullers, 2006; Paget & Trottein, 2019). Two patients in our study developed severe life‐threatening bacterial meningitis/encephalitis due to influenza. Neither of them were vaccinated against influenza or the most common bacterial meningitis agents, despite the fact that both were at risk for both influenza complications and bacterial meningitis.

There is no specific time for the onset of neurological complications as they may arise early within the first week of illness or late after the infection (Ferrari et al., 2009; Goenka et al., 2014; Paksu et al., 2018). In our study, neurological complications developed mostly within the first week. Neurological complications are associated with prolonged hospitalization, and there is a possibility for permanent neurological sequelae such as life‐long motor weakness, cognitive defects, or even death (Paksu et al., 2018). The median duration of hospitalization in our study was 10 days (ranging from 3 to 37 days). The influenza meningoencephalitic patients were treated longer at hospital compared to encephalopathic patients (15 versus 5 days). The patient with polyneuropathy was treated for the longest period of 37 days and was discharged with severe residual events, leading to disability and a significant impairment on the quality of his life.

The diagnosis of influenza‐related encephalitis remains complicated due to a lack of clear evaluating criteria. At initial presentation, blood, CSF analysis, influenza RNR in nasopharynx samples and CSF, EEG, computed tomography (CT), MRI should be performed to confirm influenza encephalopathy or encephalitis (Ambrozaitis et al., 2016; Meijer et al., 2016). The combination of rapid ELISA‐based tests with rRT‐PCR‐based diagnostic tests is especially recommended during the influenza season for diagnosing (Peteranderl et al., 2016). Up to 50%–55% of patients with IAE present normal CT scans. CT or MRI usually show lesions in the corpus callosum, white matter basal ganglia, and occasionally in the cortical and subcortical regions (Algahtani & Shirah, 2016; Ferrari et al., 2009; Goenka et al., 2014). The limitation of our study is that we did not test CSF for influenza RNR, as this method has not yet been standardized in Lithuania. It will be introduced in the future because of the real possibility of the influenza virus to cause neurological complications.

Influenza is mostly treated with antiviral drugs such as oseltamivir, zanamivir, and rimantadine. Neither of them cross the blood–brain barrier well (Ambrozaitis et al., 2016). Although the particular mechanism of antiviral therapy effect in the treatment of neurological complications is unclear, it is assumed that antiviral drugs suppress the viral expression leading to a reduction of the inflammatory response (Klein et al., 2016; Kwong et al., 2013; Paksu et al., 2018). Considering that some influenza A viruses have neurotropic properties, new antiviral drugs that cross the blood–brain barrier are needed (Davis, 2010; McCullers, 2006).

## 5 CONCLUSIONS

Our study, based on case reports, demonstrates that influenza A neurological complications are more common during pandemics. The most common neurological complication, caused by A(H_1_N_1_)2009pdm virus, was encephalopathy, affecting young patients. We speculate that it was immune mediated—related to cytokine storm. The most severe complication during pandemic was polyneuropathy with tetraparesis. The most common symptoms and signs of postpandemic (H_1_N_1_)_v_ and seasonal influenza A encephalitis were headache, dizziness, and coordination disorders. We suggest that every patient with unexplained neurological symptoms and signs similar to aseptic and septic meningitis/encephalitis should be tested for influenza virus during epidemics and pandemics. We are calling for more extensive international research on the pathogenesis, laboratory diagnosis, and treatment of influenza neurological complications.

## CONFLICTS OF INTEREST

The authors state no conflict of interest.

## AUTHOR CONTRIBUTION

Daiva Radzišauskienė is the main researcher who organized this study, participated in data collection and analysis, and wrote the manuscript. Monika Vitkauskaitė and Karolina Žvinytė wrote the manuscript. Rūta Mameniškienė revised the manuscript and supported with helpful discussions.

### Peer Review

The peer review history for this article is available at https://publons.com/publon/10.1002/brb3.1916.
